# Some Advice to Beginners

**Published:** 1912-04-15

**Authors:** Geo. W. Bowling

**Affiliations:** Lindsay, Oklahoma


### SOME ADVICE TO BEGINNERS.

BY DR. GEO. W. BOWLING, LINDSAY, OKLAHOMA.

A few words of advice to young men who are just entering the field of dentistry, and also a word of caution to those who may have backsliden or never given proper consideration to a few matters which are almost essential today.

As you have just graduated, and are about to make a start in the arena of professional life, be careful to start right. Always be punctual in meeting your appointments is probably as good a start as you can make.

A gentleman should be neat in his wearing apparel; never above all things allow himself to dress out of the ordinary business or professional style, he must not wear shoddy or loud clothes.

You and your office are your great assets. If you are not a good "mixer," stay in your office; and when you are not engaged at the chair, or in your laboratory, look around and see if there is anything which can be arranged by you that will help in keeping a tidy and clean office. If you mean to become a high-toned practitioner, here are some don'ts that should be observed;—Do not let your patient see tobacco stains on your person; and by all means never allow a cigarette stub a place in your reception room. Clean linen is to be always worn. A dentist should change shirts three times a day if necessary, rather than allow a soiled spot to be noticed by a patient, or a fellow townsman. Do not make any remarks what-so-ever, about patients for whom you worked yesterday. Never suggest to a prospective patient that so and so had work done by you last week. Some patients do not like to have it known that they are compelled to visit the dentist. Be sure that you do not let Tom, Dick and Harry, know about your private business, such as your bank account, and be careful not to make remarks about that stock you purchased last week, including that gold and silver mine in Colorado.

Be a loyal promoter of the town, county and state in which you reside. Get on the right side of every issue if possible, but do not agitate a thing just because the majority are on that side. It may be much better for your bank account if you let the other fellow do the agitating, in fact, if you want to be a politician do not open a dental office at all but study law—get yourself as soon as possible in a position to earn a livelihood from your calling .Do not by any means allow your patients to dictate to you always just the kind of work you are to do for them. Do not engage with a gossiping patient about a brother practitioner who is located just down the street from you, and has been in the same place for many years; to the contrary, go and see him after office hours, open conversation and get some pointers along the line of treating an abscessed molar or making a denture, or have a social hour with him. This will do you both good and reacts on your patients. Do not believe that because of the very fact that you are just out of college that you are up to all the latest methods, and that the old man on the other street is behind the times; the fact is, that very "hobby" of yours may have been introduced by this old practitioner ten years ago in the same school of which you are a product. The thing which hurts a great many men all over this country (business as well as professional men) is this; too many men think that because they have just learned a theorem that it is new; but, a great many times we are too self conceited; perhaps that very theorum has been propounded from many a pulpit a century ago. Perhaps the very man with whom you are discussing some subject has preached and debated on the same subject more times than you are months of age. It is always better to be on the defensive than the offensive,· even if you are right beyond the question of a reasonable doubt. Do not talk professionalisms on the street, keep far from it my lad if you would succeed. If it gets out that "that young dentist thinks he is so smart, he is always using professional language in our presence," you had better leave that country. Be modest rather than act smart. If you are not a good conversationalist you had better spend your evenings at home in your study than to press your acquaintance on your fellow townsmen.

It is better to study and plan a nice respectable office and to read your journals than to run around to moving picture shows. Do not let any one catch you loitering on the street during office hours, especially a brother practitioner, he may not be as close an observer of ethics as you, and if not he will mention the fact in the next society meeting that he looked out of his window and saw you on the street during office hours.

In conversation with any one regarding other dentists, or medical doctors, if you are asked about them critically and you do not want to commit yourself, it is better to answer them in this way, say, I do not know so and so, unfortunately my work keeps me busy and at home. I suppose so and so is a gentleman and a well informed professional man, I have not had the pleasure of meeting him yet. Be careful about making a depreciating remark about any one in their absence that you would not make in their presence, because they are sure to get it sooner or later. I am aware of the fact that several fairly good dentists whom I have known, ruined their practices in various towns by making bad remarks about their professional colleagues. It is by far the most conservative way to play "shut mouth" when any body makes an effort to bring into discussion a professional brother.

*Silence is golden. For by wise council thou shalt make thy war, and in a multitude of councilors there is safety. Proverbs 24, 6.*

*By knowledge shall the chambers be filled with all pleasant and precious riches. Proverbs 2, 4th and 24th.*

*Wisdom is too high for a fool, he openeth not his mouth in the gate. Proverbs 24, 7.*

Be ethical in that you treat others as you would have them treat you.

It is better to practice the Golden rule with your patients than to always be changing locations. *Most laymen have a fairly good knowledge of the profession, you can't fool any body.*

The most important thing is for you to hunt a good location before hanging out a sign. The city is the best place as the country towns are not inviting any more, since people are prone to go to the city if they are able to pay the price for every thing. I want to say here that you will find the best all round dentists in small and medium sized towns, but, alas; people are not willing and never will be ready to believe that kind of doctrine. The city may be the only place for you as a beginner, because nine tenths of those you work for in cities do not know but that you are as good if not the best dentist, while in the town every body knows just how much experience you have had and it is awful hard for a young man to get started in a small town. Do not locate in a small town. If you are not able to open an office in the city travel in two or three or a half dozen small towns until you you are able to open an office in the city.

You must be your own judge about when to extract a tooth or other simple operation; but don't tell a patient that you are going to amputate a root, if you do you are more than likely to be an applicant for an insane asylum the next week. *In my opinion that is where Doctor Hunter (our esteemed English false prophet) gets his fever of unknown origin.*

You will find it by far better to charge honorable fees and perhaps not do so much work than to do a whole lot of work and not get anything for it, even though you are just starting in business.

Always treat your "come back cases" as quickly as possible. If you are in doubt about an operation lasting as long as the patient expects, always tell the patient so they will not be expecting too much. Never tell a patient that a piece of bridge work will last always, rather than that tell them that it is subject to wear and tear just the same as any thing else constructed in this world by man.

It is a fact that most people expect too much at the hands of the dentist. You may use a sensible comparison and say that the filling, or whatever it may be has lasted now longer than two or a half dozen pairs of shoes will, and that it did not cost one fifth as much.

There are lots of "knockers" and they will stand in front of your office and knock until they see you are going to succeed in spite of them; then they will enter your office and want something for nothing. This is an invariable rule. Charge all such well and collect in advance. In dealing with the public from a financial standpoint, do just as the best business concerns of your city do; if the patient is known as a good bill payer, it is all right to credit him. If he is doubtful, make him pay in advance, just as the successful merchant does in carrying on his business. It is better to donate your services to those who are doubtful, than to have and hold a charge account against them; for by holding on to a bad account you make enemies and they will talk to those who do not know you, in a way to lose you practice and money. These bad bill payers are good talkers when it comes to knocking on a professional man or either a concern doing business with them. It is better if you can do a strictly cash business.

You will have lots of charity work always. Do this work just as honorably as if you were getting a good price for it, for the Good Book says,—"Blessed is he that giveth to the deserving poor." "Though I speak with tongues of men and of angles, and have not charity, I become as a sounding brass or a tinkling cymbal." Charity is every where needed and the dentist must not only possess but freely dispense it, but he should discriminate as to when and to whom he should give.

The subject of intemperance, is a very painful subject for me to comment upon. The fact that strong drink kept me down in the world for many years, makes it hard for me to call back to memory the hard times, the heart rendering episodes, and the terrible mental anguish that whiskey can produce.

I believe that from the time it issues from the coiled and poisonous worm in the distillery until it empties into the jaws of death, dis-honor, and crime, that it demoralizes every body who touches it, from its source to where it ends. It is the very life blood of the gambler, the essential element of the burglar, the prop of the highwayman, and the support of the midnight incendiary. It respects crime and defends liars; it curses God and despises Heaven. It nurses perjury and defiles the jury box. It disarms a patriot and dis-honors statesmen. It is the devil's best friend and society's worst enemy.

One cannot attain that high atmosphere in social life without holding to the highest ideals of citizenship. To be a good citizen has a broader signification than you and I are apt to give to it. Citizenship does not mean alone that to possess it one must be obedient to the law and shall be kind to his neighbour; or that he shall regard the rights of others; shall perform his duties as juror; shall if the hour of peril comes yield his time, property, and life to his country. It means also that his country shall protect him in every right that the constitution gives him. What right has a republic to demand his life and his property in the hour of peril, if when that hour of peril comes it fails in its most obvious duty?

Our profession is organized on the best democratic principles; and we must loyally support our code of ethics in spirit and letter. And we are compelled to measure up to the high calling, which it is our privilege to enjoy, and to use as one of the greatest humanitarian functions of our republic.

